# Autologous immune enhancement therapy: A case report of a stage IV colonic cancer

**DOI:** 10.3892/ol.2013.1246

**Published:** 2013-03-12

**Authors:** BASKAR SUBRAMANI, KANANATHAN RATNAVELU, CHITHRA RAMANATHAN PULLAI, KOHILA KRISHNAN, SHEELA DEVI SUGADAN, XUEWEN DENG, TERUNUMA HIROSHI

**Affiliations:** 1 Nichi-Asia Life Sciences Sdn. Bhd., Petaling Jaya 47810;; 2 Nilai Cancer Institute (NCI) Hospital, Nilai 71800, Malaysia;; 3 Biotherapy Institute of Japan, Tokyo 135-0051, Japan

**Keywords:** colonic cancer, interleukin-2, autologous immune enhancement therapy, natural killer cells, T-lymphocytes, anticancer approach

## Abstract

Current modalities of cancer treatment, including surgery, chemotherapy and radiotherapy, show marginal therapeutic responses in cancer patients. In adoptive immunotherapy, interleukin-2 (IL-2) activated immune cells demonstrated notable results in patients with advanced malignant disease. The present study reports the efficacy and safety of repetitive infusions of autologous immune enhancement therapy (AIET) in a stage IV colonic cancer patient who had already received first-line chemotherapeutic drugs. Peripheral blood was aspirated from the patient. Specifically, natural killer (NK) cells and T-lymphocytes were isolated from the peripheral blood mononuclear cells (PBMCs). These cells were activated and expanded *ex vivo* for 14 days and were transfused intravenously to the patient. After six infusions of AIET, the carcinoembryonic antigen (CEA) level was decreased from 901 to 437 U/ml, regression of lesions was noted and there were no adverse reactions during the course of this therapy. Thus, AIET may be a promising anticancer approach to eradicate tumor cells with other conventional therapies.

## Introduction

Colorectal cancer is statistically recorded as the second leading cause of mortality in Western countries and ∼50% of mortalities which occur as a result of this type of cancer are associated with progression (1). In colorectal cancer treatment, the progression-free survival rate was found to be shorter among patients with progressive metastatic colorectal cancer who were treated with chemotherapeutic drugs bevacizumab and cetuximab (CBC regimen) compared with patients who received chemotherapy and bevacizumab alone (CB regimen) (2). However, metastasis continued to result in a poor prognosis, with an average overall survival time of 20 months (3–7). The toxic side effects and symptoms experienced by patients as a result of chemotherapy have led to the development of less toxic therapies. Adoptive immunotherapy is one of the therapies and patients have experienced its benefits for the last 20 years (8).

Immunotherapy is based on the principle that the host immune system is capable of generating immune responses against cancer cells. There are plethoras of natural killer (NK) cells and antigen-induced cytotoxic T-lymphocytes (CTLs) in the human immune system. Initial efforts to generate anticancer immune responses have focused on adaptive immunity, particularly on the induction of tumor-specific CTLs. While it is appreciated that tumor-specific CTLs are critical for successful immunotherapy, it is becoming increasingly apparent that cellular components of the innate and adaptive arms of the immune system are able to control tumor growth (9).

NK cells are a component of the innate immune system, which perform immunosurveillance via the recognition of altered or missing ‘self’ surface markers in damaged, infected or transformed malignant cells (10,11). NK cells constitutively express lytic machinery that is able to kill target cells independently of any previous exposure to cancer antigens. These functional features are suggestive of their identification and control of tumor growth and metastasic diffusion *in vivo*.

The main functions of NK cells are to suppress tumor cell initiation, growth and metastasis through mechanisms mediated by perforin and the granzyme-containing granule-mediated death receptor and interferon-γ-mediated pathways (12). These varied functions of NK cells hold considerable potential for cell-based therapies which target human malignancies (11,13–16).

Progress in immunobiotechnology, following extensive investigation, has permitted clinical trials with *in vitro* derived NK cells and CTLs, which may be adoptively transferred to patients via a painless procedure for cancer treatment (17).

Autologous immune enhancement therapy (AIET) has had a successful clinical history in Japan, Europe and the USA over the past two decades. Immune cell therapy using autologous activated lymphocytes (18,19) was first introduced in the laboratory by Rosenberg *et al* of the National Institute of Health, USA. In the late 1980s, Rosenberg *et al* published a key study which reported a low tumor regression rate (2.6–3.3%) in 1,205 patients with metastatic cancer who had undergone different types of active specific immunotherapy (ASI).

In the present study, we report the results from a colorectal cancer patient who underwent 6 infusions of immune cell therapy.

## Case report

### Patient history

A 50-year-old female diagnosed with stage IV colonic cancer in May 2008 presented with a *KRAS* mutation. CT scans revealed a malignancy in the liver and the carcinoembryonic antigen (CEA) level was 500 U/ml. The patient was administered FOLFOX-6 between June 2008 and September 2008. The disease progressed and selective internal radiation therapy (SIRT) was administered. After the therapy, the patient remained healthy for 2 months and the CEA level began to increase. Thus, Bevacizumab and FOLFIRI was restarted between November 2008 and April 2009. The patient was unable to tolerate the chemotherapeutic side effects and opted to receive AIET.

### Ex vivo expansion of NK cells and T-lymphocytes (TLs) from the peripheral blood

**Figure 1:**
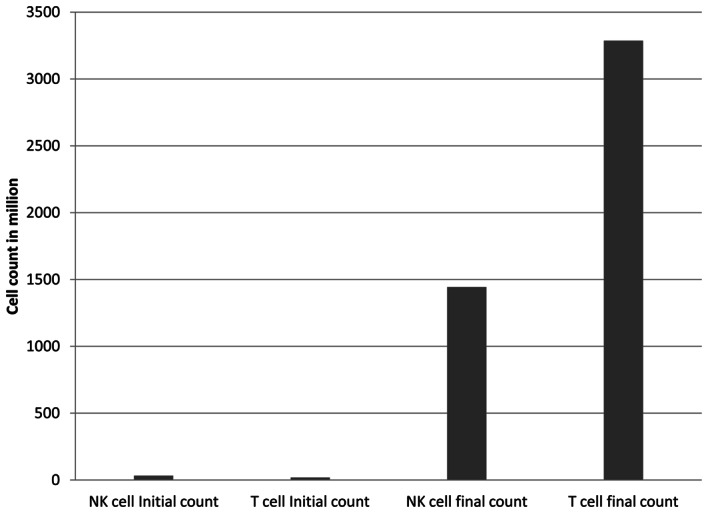
Comparison of the total numbers of NK cells and T-lymphocytes before and after cell expansion for all 6 infusions. NK, natural killer.

After informed consent was obtained from the patient, 60 ml peripheral blood was withdrawn. The activated NK cells and TLs were isolated from the peripheral blood mononuclear cells (PBMCs) of the patient, and later were activated and expanded according to previously described methods (17), for 14 days. The average number of PBMCs before the six times of expansion was 32.7×10^6^ and 19.5×10^6^ for NK cells and TLs, respectively. After 2 weeks of expansion, the average number of expanded lymphocytes for six injections was 14.4×10^8^ for NK cells and 32.9×10^8^ for TLs. The average frequency of NK (CD3^−^/CD56^+^) cells was 6.3% initially and 40.5% at final culture. For TLs (CD3^+^), the average frequency was 88.9% initially and 62.1% at final culture, as evaluated by flow cytometry. The total infused cell numbers are shown in Fig. 1. Before cells were infused, a sterility test, using bacterial and fungal agar plates, and an endotoxin test using Limulus amoebocyte lysate (Wako, Tokyo, Japan) were carried out to confirm the asepsis of the products.

### Effects of AIET

**Figure 2:**
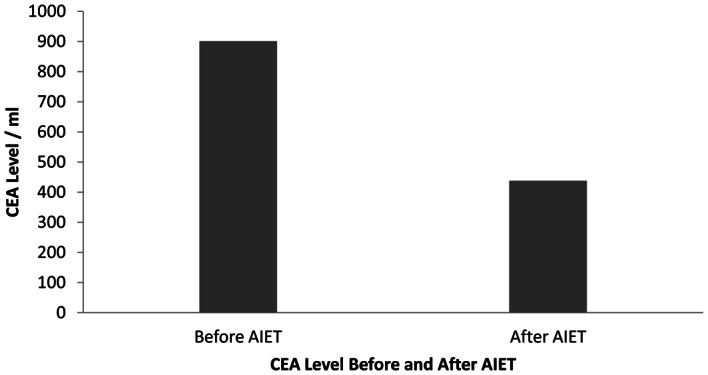
Comparison of CEA level before and after AIET. CEA, carcinoembryonic antigen; AIET, autologous immune enhancement therapy.

**Figure 3:**
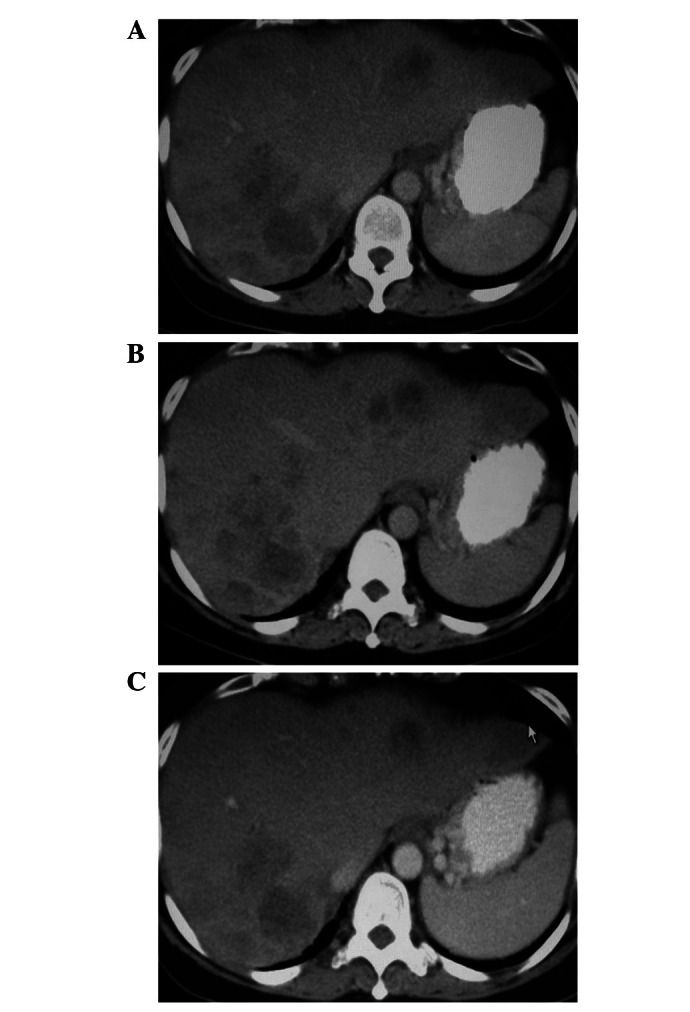
CT scans showing regression of the lesion following AIET infusions. (A) Lesion before infusion, (B) after the third infusion and (C) after the sixth infusion. AIET, autologous immune enhancement therapy.

After 6 infusions of NK cells and TLs, the CEA level decreased considerably from 901 to 437 U/ml (Fig. 2). The CT scan revealed that the patient with distant metastases (stage IV) responded to the treatment with tumor reduction in one liver lobe. Tumor assessment was carried out after the third and sixth infusion. The lesion in the colon and lung (not shown) was stable; however, the size of the liver metastasis was markedly reduced (Fig. 3).

## Discussion

In this study, we evaluated the safety and efficacy of the *ex vivo* adoptively transferred, activated and expanded NK cell s and TLs derived from the peripheral blood of a colorectal cancer patient. The patient was followed up for 8 months from the first infusion of AIET. CEA levels were recorded before and after injection of the AIET (Fig. 2). The average initial and final cell counts were recorded at the time of each sample collection and expansion (Fig. 1).

In this course of treatment, interleukin-2 (IL-2) was not administered. Following standard procedures, the effector cells were cultured with IL-2, which ranged from 350 to 700 IU/ml over the duration of 0–14 days. For all six infusions, the fold expansion of lymphocytes from PBMCs was ∼44 and 168 for NK cells and TLs, respectively. With regard to this fold expansion, it was concluded that the total number of activated and expanded cells are highly dependent on the initial lymphocyte population of PBMCs of the whole blood. Notably, inverted phase contrast microscopy (data not shown) revealed that cell viability and the frequency of the healthy population was clear in each sample processing during the culture of PBMCs.

The expanded cells were administered intravenously. We determined that the infusion of heterogeneous TL and NK cell populations induced a decrease in tumor mass in this case, as shown by the CT scan results (Fig. 3C). We concluded that the acceleration of tumor growth was arrested by the infused effector cells, since there were no other supportive therapies administered with or in between AIET. This was further supported by a testimonial from the patient which described improved physical strength and an active lifestyle.

Several case reports demonstrate that CTLs and NK cells improve the cytotoxic effects in cellular immune responses to similar levels as those identified in *in vitro* studies (20–23). NK cell infiltration is an indicator in patients with colorectal carcinoma. Colorectal carcinoma displays ligands for the activating receptors, loss of HLA class I molecules and dismissed MHC class I, which shows the susceptibility for NK cell-mediated lysis (11,24).

CTLs also play a critical role in the adaptive immune system. CTLs and NK cells secrete granzyme and perforin, which are packaged in cytoplasmic granules (25) to lyse cancerous cells. For a decade, it has been widely accepted that CD8^+^ T cells are correlated with an effective antitumor response (26), patient survival (27) and the control of tumor invasion and metastasis (28).

A number of studies support the sensitivity of CTLs against colorectal cancer *in vitro* and *in vivo*. Todaro *et al*(29) showed that colon cancer stem cells were sensitive to γδT cells, a small subpopulation of TLs which have the ability to target cancerous cells. It has been hypothesized that tumor reduction and an improved quality of life may have been derived from the presence of *ex vivo* derived CTLs and γδT cells. CTLs and γδT cells kill their targets via the secretion of perforin and granzyme B, which supports the clinical observations in the present case.

However, the cancer stem cell population and its dysfunction in the presence of CTLs and γδT cells requires further investigation in clinical studies. Due to the minor population of γδT cells *in vivo*, whether γδT cells are able to effectively recognize cancer cell populations is questionable. This has been clarified by a large cohort study. Ogino *et al*(30) showed that lymphocytic reactions to tumors were associated with longer survival in colorectal cancer patients. Although the study did not thorougly analyze the subpopulations of inflating lymphocytes, it confirms the importance of immune targeting therapies.

Numerous studies which explored NK cells and TLs have demonstrated the impact of immune contexture in human tumors and its clinical outcome. In a study involving colon cancer patients with a history of surgery, 20–25% of patients had recurrence of their disease, suggesting that occult metastases are already present at the time of surgery (31). Adjuvant therapy contributed to the survival of 86.2% of patients after 5 years, whereas 72% of patients with a low immune score had disease recurrence and only 27.5% of these survived after 5 years (32). Thus, immune enhancement may be a solution with regard to improving survival rates.

These results encourage most attempts at cancer immunization, which directly involve *ex vivo* clonal expansion of CD8^+^ T cells and CD4^+^ T cells to activate and endure CD8^+^ killer cells (33). A number of studies note that the infusion of *ex vivo* derived therapies are effective with a reasonable dose of IL-2 or other combined therapies (34,35). Although the administration of IL-2 provided successful outcomes, its toxicity could not be tolerated by patients and in certain cases, patients succumbed to this toxicity (36).

In conclusion, considering the immune contexture, CTLs and NK cells may be useful tools for the benefit of patients by enhancing their internal tumor infiltrating lymphocytes. Adoptive therapy demonstrated long-term disease-free survival, a notable improvement in quality of life and also an improved overall survival and therefore, may be recommended to patients with no other treatment options. Although AIET has been shown to benefit patients, longer follow-up is required before a final conclusion may be determined.
